# A functional description of adult picky eating using latent profile analysis

**DOI:** 10.1186/s12966-018-0743-8

**Published:** 2018-11-06

**Authors:** Jordan M. Ellis, Hana F. Zickgraf, Amy T. Galloway, Jamal H. Essayli, Matthew C. Whited

**Affiliations:** 1 Department of Psychology, East Carolina University, East Fifth Street, 104 Rawl Building, Greenville, NC 27858-4353 USA; 2 Department of Psychiatry and Behavioral Neuroscience, University of Chicago, 5841 S. Maryland Ave, Chicago, IL 60637 USA; 3 Department of Psychology, Appalachian State University, 222 Joyce Lawrence Ln, P.O. Box 32109, Boone, NC 28608 USA; 4 Department of Psychiatry, Pennsylvania State University College of Medicine, 500 University Drive, Hershey, PA 17033 USA

**Keywords:** Adult picky eating, Latent profile analysis, Eating behavior, Body mass index

## Abstract

**Objective:**

Research has indicated that adult picky eating (PE) is associated with elevated psychosocial impairment and limited dietary variety and fruit and vegetable intake; however, research operationalizing PE behaviors is limited. Previous research identified a PE profile in children, marked by high food avoidance (satiety responsiveness, fussiness, and slow eating) and low food approach (food enjoyment and responsiveness) appetitive traits. The present study aimed to replicate a similar latent eating behavior profile in an adult sample.

**Methods:**

A sample of 1339 US adults recruited through Amazon’s MTurk completed an online survey that included a modified self-report version of the Child Eating Behavior Questionnaire (CEBQ-A). Latent profile analysis was employed to identify eating profiles using the CEBQ-A subscales, ANCOVAs were employed to examine profile differences on various self-report measures, and eating profiles were compared across BMI classifications.

**Results:**

Analyses converged on a four-profile solution, and a *picky eater* profile that closely resembled the past child profile emerged. Participants in the *picky eater* profile (18.1%) scored higher on measures of adult PE and social eating anxiety compared to all other profiles, scored higher on eating-related impairment and depression than moderate eating profiles, and were more likely to be of normal weight.

**Discussion:**

A distinct adult PE profile was observed, indicating childhood PE and appetitive behaviors may carry over into adulthood. Research identifying meaningful groups of picky eaters will help to shed light on the conditions under which picky eating is a risk factor for significant psychosocial impairment or distress, or weight-related problems.

## Introduction

Picky eating (PE) is typically characterized as eating from a narrow range of food, rigidity about how preferred foods are prepared or served, and difficulty trying novel foods [1, 2] PE is common in childhood and adulthood, with relatively wide-ranging estimates converging on a prevalence of 15–35% across the lifespan [2–4]. Although not defined as a form of disordered eating, adult PE is associated with eating-related psychosocial impairment and anxiety [5, 6]. PE is also one pattern of restrictive eating that can result in symptoms of Avoidant/Restrictive Food Intake Disorder (ARFID), a diagnosis new to the fifth edition of the Diagnostic and Statistical Manual [7]. ARFID is designed to identify individuals of any age whose restrictive eating leads to inadequate caloric intake and/or dietary variety, resulting in weight loss, nutritional deficiency, dependence on nutritional supplements, and/or psychosocial impairment [7]. Research on PE in both children and adults has been limited by inconsistent and varied approaches to measurement, which reduces researchers’ ability to understand and compare correlates and outcomes across samples [2, 6]. Many past studies have relied on a single self-report or parent-report item to assess PE [2, 8]. Other researchers have developed short subscales to assess fussy/picky eating [5, 9, 10], though items from these measures tend to focus on food neophobia and limited food variety and may not be capturing other important aspects of PE behaviors and attitudes. Recent work has led to the development of a multidimensional measure of adult PE, which aimed to capture rigid preferences related to food presentation, aversions to specific tastes, and avoidance of mealtime that are common to PE [6]. While efforts to improve the measurement of PE are hopeful, more work is needed to further operationalize the construct as a means of improving the comparability of findings and understanding what aspects of PE or PE subgroups differentiate amongst health and psychosocial outcomes.

Variability in measurement methods likely contributes to inconsistent and unclear findings regarding the health impact of PE. For example, although across the lifespan PE is consistently associated with lower fruit and vegetable intake and variety [8, 11, 12], which is typically associated with higher weight in adults (prospective studies also link higher fruit and vegetable consumption to lower risk of weight gain and obesity), picky eating is not cross-sectionally associated with overweight in children [13] or with BMI in adults [3, 6, 10, 14]. In childhood, picky eating may actually be a long-term protective factor against the development of overweight [15, 16] and in a recent meta-analysis, approximately half of cross-sectional studies identified reported slight inverse correlations with BMI in children ages 0–18, with the remainder reporting a null association [13]. The conditions and contexts under which picky eating might contribute to over vs. underweight or be protective against overweight in adulthood are not well understood. Given PE’s association with eating behavior that should increase obesity risk, and commonality across the lifespan and weight spectrum [2–4], it would be useful to understand how picky eating may combine with other eating behaviors in adulthood to predict differential weight outcomes. Similarly, although both childhood and adult picky eating have been linked to anxiety and depression symptoms [17, 18], and adult picky eating to eating-related clinical impairment [5, 19], these findings may be attenuated when other restrictive eating behaviors are statistically controlled [20]. As research on the phenomenology and treatment of ARFID develops and is extended into adult samples, there is a need to better understand the conditions and contexts under which picky eating does and does not lead to weight/nutritional problems and/or psychosocial impairment.

One approach to explore differential weight and psychosocial outcomes and further operationalize PE is to understand how PE clusters with other appetitive traits. Latent profile analysis has been used to identify a combination of appetitive traits (including picky eating) that is concurrently and prospectively associated with low weight in young children [21, 22]. This *“fussy”* eating profile was characterized by high levels of picky eating, satiety responsiveness, and slow eating, as well as low levels of food enjoyment and food responsiveness [22]. Along with being more likely to be underweight, children identified as *“fussy”* had eaten fewer servings of whole grains, vegetables, fish, and meat than other children, but more servings of sweets and snack foods [22]. In another cohort of young children, Sandvik and colleagues (2018) found that 17% of preschool-aged children with overweight or obese BMI were identified as picky eaters, and these children had higher parent-reported food responsiveness compared to healthy- or underweight picky eaters [23]. These findings demonstrate that considering adult PE in the context of other appetitive traits may reveal important subgroups of adult picky eaters and clarify relationships with important health and psychosocial outcomes.

Although much of the previous research on appetitive traits has been conducted in children, there is considerable convergence in findings from the childhood literature and the relatively limited number of findings from adult samples on the relationships between appetitive traits and BMI [14], the relationship between picky eating and dietary intake/variety [8, 24], and the relationships among the appetitive traits [10, 14]. To date, the stability of the appetitive traits from childhood to adulthood has not been studied, with the exception of picky eating, which has been shown to persist across childhood [18] and into young adulthood [25]. Other appetitive traits have shown stability from birth to 10 years old [21, 26, 27]. In addition, all of these traits show evidence of high heritability, suggesting that they are best thought of as stable traits that are expressed relatively similarly across the lifespan [28–30]. Given the relationship of combinations of childhood appetitive traits to weight and nutritional outcomes both cross-sectionally [22] and longitudinally [21], there is a need for further study of how appetitive traits relate to each other and to relevant outcomes in adulthood.

The primary aim of the present study was to determine if a similar latent eating behavior profile previously identified in children using a measure of food approach/avoidance traits is also observed in a general adult sample. It was hypothesized that a picky eating profile would emerge, consisting of low scores on food approach (food responsiveness and enjoyment of food) and high scores on food avoidance (satiety responsiveness, food fussiness, and slowness in eating), reflecting the *fussy eater* profile identified in a sample of children by Tharner and colleagues (2014) [22]. The second aim of the study was to provide convergent validity for an adult *“picky eater”* profile by exploring differences among the other emergent profiles on measures of adult PE, psychosocial impairment, intuitive eating, and self-reported BMI. It was hypothesized that individuals classified in the *picky eater* profile would score higher on a measure of adult PE, and, consistent with previous research on distress and impairment, it was predicted that individuals in the *picky eater* profile would report greater depressive symptoms, social eating anxiety, and eating-related impairment, compared to other eating presentations. It was predicted that picky eaters would be more likely than individuals in other profiles to eat due to biological cues (hunger and satiety), and less likely to eat in response to external food cues or emotions, through measures of intuitive eating. Finally, we expected to replicate Tharner and colleagues’ (2014) finding that individuals in the *picky eating* profile would report lower BMI.

## Method

### Procedure

A total sample of 2187 individuals participated in an online survey about through Amazon’s Mechanical Turk (MTurk). MTurk is an online platform where “workers” can be paid by “requesters” to perform small tasks. Called human intelligence tasks, or HITs, these tasks were originally ones that computers were unable to complete, such as interpreting blurry images or providing feedback on the usability of corporate websites. The platform is also widely used to collect survey data for psychological research [31]. Workers view a brief survey description and the payment amount, before agreeing to complete the HIT (i.e., complete the survey). The present survey was described as follows: *The purpose of this survey is to examine different aspects of growth and development in US adults*, and participants were given no indication the survey included questions about eating behaviors. Participants informed that the survey would take approximately 10–15 min, which is how long it took research assistants to complete the survey quickly without making mistakes. Participation was restricted to US workers. To ensure quality responses, MTurk worker qualifications/requirements to participate included a HIT approval rate of greater than 80% (# of approved HITs that a worker has completed) and number of HITs approved greater than 1000 (# of HITs that a worker has successfully completed since registering with MTurk). Validity items were embedded throughout the survey (e.g. “Please select *Often* as the response to this item”), and participants were automatically excluded if they did not correctly respond to the validity items. Participants were paid $0.50 for completing the survey, making participation essentially voluntary. Potential participants were informed of the compensation and predicted time duration prior to starting the task. The online survey first obtained informed consent and confirmed that participants were 18 or older. Appalachian State University’s Institutional Review Board approved the study’s procedure.

### Measures

#### Demographics and anthropometrics

Participants responded to several demographic questions assessing gender, age, race/ethnicity, and education level. They also self-reported their height and weight.

#### Child eating behavior questionnaire-adult version

A modified version of the Child Eating Behavior Questionnaire (CEBQ was administered to assess food approach and avoidance behaviors [9]. The CEBQ, which has been used in previous research as a parent-report to measure the degree of individual differences in the eating styles of children, was modified and used as a self-report in this adult sample. The modified items were similar or identical to items from the newly developed Adult Eating Behavior Questionnaire (AEBQ) [10]; though the AEBQ had not been validated at the time of data collection. The AEBQ subscales have since demonstrated good internal consistency (*α’s*: 0.75 to 0.90) and 2 week test-retest reliability (ICCs: 0.73 to 0.91) [10]. Examples of modified items, assessed on a five-point Likert scale, included: “I enjoy tasting new foods” and “I am interested in tasting foods I haven’t tasted before.” Five of the eight CEBQ subscales were used in this study: Food responsiveness, enjoyment of food, satiety responsiveness, slowness in eating, and food fussiness. Following Tharner’s (2014) methodology, the emotional over-eating and emotional under-eating scales were excluded to capture general food approach and avoidance as opposed to emotional eating behaviors, and the desire to drink scale was excluded because its face validity was questionable in an adult sample, who might interpret the items to refer to drinking alcohol [10]. The five remaining subscales demonstrated good internal consistency (*α*_*Food* Responsivenss_ = .87; *α*_*Enjoyment*_ = .83; *α*_*Satiety* Responsiveness_ = .70; *α*_*Fussy*_ = .86; *α*_*Slowness*_ = .84).

#### Adult picky eating

The Adult Picky Eating Questionnaire (APEQ) is a validated 16-item self-report scale that assesses PE behaviors and attitudes in adults [6]. Participants respond to items that describe eating behavior on a 5-point Likert scale from 1-“never” to 5-“always.” A composite score is calculated by averaging the 16 items. The APEQ demonstrated good internal consistency in the present study (*α* = .89).

#### Social eating anxiety

Social eating anxiety as measured by a questionnaire developed by Wildes and colleagues (2012) has a demonstrated positive association with adult picky eating [5]. The scale includes three items measuring anxiety around social situations involving food and eating, using a five-point Likert scale from 1-“rarely or never” to 5-“all the time.” Composite scores are summed and range from 3 to 15, with higher scores indicating greater anxiety. The scale demonstrated good internal consistency in the present study (*α* = .91).

#### Depressive symptoms

Depressive symptoms were assessed using the Patient Health Questionnaire-9 (PHQ-9) [32]. The PHQ-9 has demonstrated good reliability and validity for use in clinical and research settings [32]. Participants rated each of the nine DSM-IV criteria for depression on a scale from 0-“not at all” to 3-“nearly every day”, and the scores were then summed. The PHQ-9 demonstrated good internal consistency in the present study (*α* = .92).

#### Eating-related quality of life

The Clinical Impairment Assessment questionnaire (CIA) is a 16-item self-report measure that was developed to assess psychosocial impairment due to disordered eating [33], and is rated on a 4-point Likert scale ranging from 0-“not at all” to 3-“a lot.” Items were summed for a composite score. The measure was modified to capture responses about both disordered and picky eating by asking participants, “Over the past 28 days, to what extent have your eating habits or concerns about your eating…” as opposed to asking about exercise and feelings about shape or weight that are exclusive to traditional eating disorders. This modification has been used in other studies investigating adult PE and ARFID [4, 5]. In the current study the CIA demonstrated good internal consistency (*α* = .96).

#### Disordered eating symptoms

The Eating Disorder Diagnostic Scale (EDDS) was utilized to assess for disordered eating behaviors and symptoms. It is a 22-item measure that has demonstrated evidence for good reliability and validity and can be used to support clinical diagnoses [34]. Because this study did not use a clinical sample, a continuous eating disorder symptom composite score was calculated by summing the raw items to assess symptom severity [35]. The EDDS composite score demonstrated satisfactory internal consistency in the present study (*α* = .71).

#### Intuitive eating

The Intuitive Eating Scale (IES) is a 21-item questionnaire developed to serve as a measure for adaptive eating that consists of three subscales comprised of seven items each: unconditional permission to eat (which assesses the absence of restraint), eating in response to emotions, and eating in response to signs of hunger and satiety [36]. Participants responded to items on a 5-point Likert scale ranging from 1-“strongly disagree” to 5-“strongly agree,” with higher scores indicating more intuitive eating/less restraint and emotional eating. Items were summed and averaged for each subscale The IES has demonstrated good construct validity and test-retest reliability [36]. The IES subscales demonstrated good internal consistency in this study (*α*_*Unconditional*_ = .83; *α*_*Emotional =*_ .90; *α*_*Hunger*_ = .81).

### Data analysis

Latent profile analysis (LPA) was employed to identify eating behavior profiles using CEBQ-A subscales that had been standardized to z-scores. LPA analyses were conducted in R [37], using the mclust package [38]. LPA identifies clusters of observations across similar values on continuous variables and then models profiles based on subject responses [38]. Following methods described by Tharner and colleagues (2014), five scales from the CEBQ-A (food responsiveness, enjoyment of food, satiety responsiveness, slowness in eating, and food fussiness) were used in the LPA. The number of emergent latent profiles was based on the minimization of the Bayesian information criteria (BIC) and the sample-size adjusted BIC (aBIC). BIC and aBIC approaching 0 indicates the best model fit. Simulated models have indicated that the BIC and aBIC are good at selecting the correct number of classes in eating behavior research [39]. Subjects were assigned to profiles based on Bayesian probabilities after determining the optimal number of eating behavior profiles.

To determine possible covariates for subsequent analyses, Pearson Chi-square analyses and post-hoc Bonferroni adjusted z-tests to compare column proportions were employed to examine demographic differences (i.e. age, gender, race/ethnicity, completed level of education, and estimated family income) among the latent profiles. Race/ethnicity (White and nonwhite), education (< 4-year college degree and ≥ 4-year college degree), and income (< $50,000 and ≥ $50,000) were dichotomized. Categorical group differences between profile groups and BMI classification (i.e. underweight, normal weight, overweight, and obesity) were also tested. Latent profile characteristics were then examined using SPSS 24.0. Bootstrapped Analyses of Covariance (ANCOVAs), using 1000 bootstrapped samples and controlling for age, gender, and education, were employed to examine group differences on continuous variables, and post hoc comparisons amongst profile groups were also calculated. The moderate eating profile was used as the reference group. Outcome variables examined using ANCOVAs included: self-reported BMI, the EDDS, PHQ-9, social eating anxiety, the APEQ composite score and subscales, and the IES composite and subscales. Given the large sample and large number of post hoc comparisons conducted subsequent to the ANCOVAs, Bonferroni corrections and a more conservative *p*-value of *p* < .01 were employed.

## Results

Of the 2187 MTurk workers who participated in this study’s HIT, 546 (25.0%) dropped out before completing the survey and an additional 302 failed embedded validity checks, resulting in a final sample of 1339 participants. As shown in a recent systematic review, more than 20% of MTurk studies have high dropout rates (> 30%) [40]. It is thought that workers will enter a HIT and dropout if they have disinterest in the nature of the task or seek greater compensation from another HIT, which results in self-selection [41]. The median completion time was 15.46 min. Completion time ranged from 3 to 72 min. Fewer than 25 participants completed in under 5 min and 75% of participants completed in under 20 min. The upper end of the distribution likely reflects individuals who may have stepped away from the survey and left it open on their web browser. While the attention of those who complete in less than 5 min is questionable, they did pass the validity checks and a visual inspection of the data did not indicate distorted responding. The final sample includes 804 women and 535 men, and the race/ethnicity distribution was 80% White, 10% Black, 5% Asian, 3% Hispanic. The mean age of the sample was 40.39, SD = 13.39. The mean self-reported BMI of the sample was 27.67 (SD = 7.18), and 53.0% of the sample reported completing at least a 4-year college degree. See Table 1 for a full description of the demographic characteristics of the participants.

**Table 1:** Demographic Characteristics of the Participants (*n* = 1339)

Variable	Mean	SD
Age	40.39	13.39
BMI	27.67	7.18
CIA	7.35	9.60
PHQ-9	5.46	6.04
Social Eating Anxiety	4.35	0.99
EDDS (standardized)	0.00	0.61
APEQ Total	2.30	0.68
IES-Uncond	3.19	0.77
IES-Phys	3.24	0.96
IES-Hung	3.69	0.63
Variable	*n*	Percentage
Gender (Female)	804	60.0
Income		
Less than $20,000	207	15.5
$20–35,000	295	22.0
$35–50,000	292	21.8
Over $50,000	545	40.7
Education		
< High School/GED	5	0.4
High School/GED	146	10.9
Some College	311	23.2
2-year College Degree	167	12.5
4-year College Degree	505	37.7
Master’s Degree	151	11.3
Doctoral Degree	24	1.8
Professional Degree	20	1.5
Technical or Vocational School	10	0.7
Race/Ethnicity		
White	1070	79.9
Black	129	9.6
Hispanic	46	3.4
Asian	66	4.9
Native American	10	0.7
Pacific Islander	1	0.1
Other	17	1.3

*Note: BMI* Body mass index, *CIA* Clinical impairment assessment, *PHQ-9* Patient health questionnaire - 9-item, *EDDS* Eating disorder diagnostic scale, *APEQ* Adult picky eating questionnaire, *IES-Uncond* Intuitive eating scale- Unconditional permission to eat scale, *IES-Phys* Eating for physical vs. emotional reasons scale, *IES-Hung* Eating in response to hunger/satiety scale

### Latent profile analysis

**Fig. 1:**
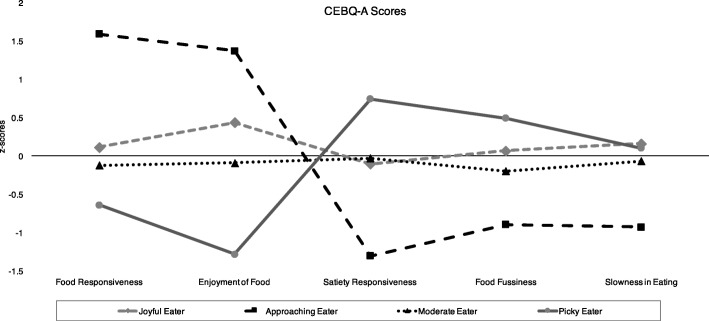
CEBQ-A mean subscale scores (z-standardized) in different eating behavior profiles. Child eating behavior questionnaire – Adult self-report version (CEBQ-A) mean subscale scores (z-standardized) for the four eating behavior profiles identified through latent profile analysis. Participants grouped in the *Joyful Eater* profile reported moderate scores on most subscales, but higher food enjoyment compared to the *Moderate Eater* profile. Respondents in the *Approaching Eater* profile reported high food approach (i.e. food responsiveness and enjoyment of food) and low food avoidance traits. Participants grouped in the *Picky Eater* profile reported low food approach and high food avoidance (i.e. satiety responsiveness and food fussiness)

LPA indicated that the BIC minimized at five profiles (BIC = 17,286.01) and the aBIC minimized at four profiles (SABIC = 17,473.66). Thus, the four- and five-profile solutions were examined in relation to the profiles modeled by Tharner (2014). The four-profile solution provided four profiles that were distinct on food approach and avoidance traits, and closely resembled four of the six profiles (“moderate,” “picky,” “joyful,” and “approaching”) previously identified in children [22]. The five-profile appeared to split a low approach/high avoidance profile into two non-distinct profiles. Further considering that simulations have shown the SABIC to provide the best overall performance as an information criteria when applied to eating disorder research [39], it was concluded to proceed with a 4-profile solution. Figure 1 shows the pattern of CEBQ-A scores for each of the four identified eating behavior profiles, including a distinct *“picky eater”* profile. The picky eating profile (18.1% of participants) was characterized by a pattern of low scores on the food approach subscales (food responsiveness and enjoyment of food), high scores on two of the food avoidance subscales (satiety responsiveness and food fussiness), and moderate scores on the slowness in eating subscale.

The remaining three profiles were: 1) a *“moderate eater”* profile (34.6% of participants), characterized by scores close to the mean on all subscales; 2) a *“joyful eater”* profile (39.9%), characterized by scores near the mean on food avoidance food scales, scores above the mean on enjoyment of food, and average food responsiveness; and 3) an *“approaching eater”* profile (7.5%), with high scores on food approach subscales and low scores on food avoidance subscales.

### “Picky eater” profile characteristics

Given the aims of this study, the ANCOVA results reported below focus on differences between the *picky eater* profile and each of the other profiles. However, comparisons between all four eating behaviors profiles are noted in Table 2. Chi-square analyses indicated differences in education, BMI classification, and age among the latent groups, thus they were included as covariates in subsequent analyses (See Table 3). Gender was also included as a covariate, as female participants reported more disordered eating (*r*_*EDDS*_ = .10, *p* < .001), and less intuitive eating (*r*_*IES_Uncond*_ = −.08, *p* = .003; *r*_*IES_Phys*_ = −.09, *p* = .001) and PE (*r*_*APEQ*_ = −.11, *p* < .001) compared to male participants. As shown in Table 4, ANCOVAs, controlling for age, gender, education, and BMI (except for the BMI ANCOVA), indicated that there were significant differences among eating behavior profile adjusted means on all outcome variables except for the IES Unconditional Permission to Eat profile. Observed and adjusted means are reported in Table 2, and the shows pairwise comparisons between the *picky eater* profile and other three profiles on all outcome variables. Participants grouped in the *picky eater* profile self-reported lower BMI scores (adjusted mean = 26.04) in comparison to *joyful eater* (adjusted mean = 27.94), and *approaching eater* (adjusted mean = 30.76) profiles, but not the *moderate eater* (adjusted mean = 27.53) profile.

**Table 2:** Characteristics of the latent eating profiles

	Observed Mean	SD	Adjusted Mean	se
Self-reported BMI				
Moderate	27.61	6.95	27.53_a, b_	0.33
Picky	25.89	6.89	26.04_a_	0.46
Approaching	30.51	8.46	30.76_c_	0.71
Joyful	27.99	7.06	27.94_b_	0.32
CIA				
Moderate	5.60	7.59	5.82_a_	0.42
Picky	9.14	10.73	9.36_b_	0.59
Approaching	10.73	10.56	9.23_b_	0.92
Joyful	7.45	10.12	7.44_b_	0.39
PHQ-9				
Moderate	4.53	5.06	4.65_a_	0.27
Picky	7.46	6.71	7.60_b_	0.37
Approaching	6.88	7.12	6.08_a, b_	0.58
Joyful	5.13	6.06	5.12_a_	0.25
Social Eating Anxiety				
Moderate	4.41	2.33	4.46_a_	0.13
Picky	6.09	3.44	6.10_c_	0.19
Approaching	4.55	2.72	4.25_a, b_	0.29
Joyful	5.01	3.13	5.02_b_	0.12
EDDS				
Moderate	−0.08	0.54	−0.07_a_	0.03
Picky	−0.03	0.64	−0.00_a_	0.04
Approaching	0.37	0.59	0.26_b_	0.06
Joyful	0.01	0.62	0.01_a_	0.02
APEQ				
Moderate	2.15	0.54	2.15_b_	0.03
Picky	2.64	0.68	2.65_d_	0.04
Approaching	1.91	0.51	1.86_a_	0.07
Joyful	2.35	0.73	2.36_c_	0.03
IES-Uncond				
Moderate	3.20	0.67	3.20_a_	0.04
Picky	3.18	0.82	3.17_a_	0.05
Approaching	3.07	0.78	3.11_a_	0.08
Joyful	3.19	0.82	3.20_a_	0.03
IES-Phys				
Moderate	3.28	0.78	3.27_b, c_	0.04
Picky	3.52	0.99	3.47_c_	0.06
Approaching	2.54	1.07	2.68_a_	0.09
Joyful	3.21	1.00	3.22_b_	0.04
IES-Hung				
Moderate	3.67	0.48	3.67_a_	0.03
Picky	3.60	0.71	3.58_a_	0.04
Approaching	3.44	0.82	3.49_a_	0.07
Joyful	3.81	0.64	3.81_b_	0.03

*Note:* Subscripts that differ represent significant pairwise differences between the profiles (*p* < .01). The Bonferroni correction for pairwise comparisons was employed. Means are adjusted to control for age, gender, education, and BMI in all but the BMI comparison. Results based on 1000 bootstrapped samples

*BMI* Body mass index, *CIA* Clinical impairment assessment, *PHQ-9* Patient health questionnaire - 9-item, *EDDS* Eating disorder diagnostic scale, *APEQ* Adult picky eating questionnaire, *IES-Uncond* Intuitive eating scale- Unconditional permission to eat scale, *IES-Phys* Eating for physical vs. emotional reasons scale, *IES-Hung* Eating in response to hunger/satiety scale

**Table 3:** Demographic and clinical characteristics of the latent profiles

	Moderate	Picky	Approaching	Joyful	Test Statistic
Gender (Female)	268 (57.9%)_a_	157 (64.9%)_a_	57 (57.0%)_a_	322 (60.3%)_a_	*Χ* ^*2*^(3) = 3.65
Nonwhite race/ethnicity	88 (19.0%)_a_	43 (17.8%)_a_	24 (24.0%)_a_	114 (21.3%)_a_	*Χ* ^*2*^(3) = 2.63
≥ 4-year college degree	307 (66.3%)_a_	139 (57.4%)_a,b_	51 (51.0%)_b_	350 (65.5%)_a_	*Χ* ^*2*^(3) = 13.04**
≥ $50,000 estimated family income	209 (45.1%)_a_	93 (38.4%)_a_	44 (44.0%)_a_	199 (37.3%)_a_	*Χ* ^*2*^(3) = 7.36
BMI Classification					*Χ* ^*2*^(9) = 39.81***
Underweight (BMI < 18.5)	9 (2.0%)_a_	11 (4.6%)_a_	3 (3.1%)_a_	10 (1.9%)_a_	
Healthy weight (BMI = 18.5–24.9)	187 (40.7%)_a_	125 (52.3%)_b_	29 (29.6%)_a_	205 (38.9%)_a_	
Overweight (BMI = 25–29.9)	141 (30.7%)_a_	57 (23.8%)_a_	21 (21.4%)_a_	154 (29.2%)_a_	
Obese (BMI ≥ 30)	123 (26.7%)_a,b_	46 (19.2%)_b_	45 (45.9%)_c_	158 (30.0%)_a_	
	*M* (SD)	*M* (SD)	*M* (SD)	*M* (SD)	
Age	41.49 (12.99)_a_	38.03 (13.24)_b_	36.91 (11.05)_b_	41.07 (13.97)_a_	*F* (3,1335) = 6.54***

*Note:* Each subscript letter denotes a subset of latent eating profile groups whose column proportions do not differ significantly from each other based on Bonferroni adjusted *p*-value. ** *p* < .01, *** *p* < .001

**Table 4:** ANCOVAS for body mass index, psychosocial impairment, and eating behaviors

Scale	Predictor	*F*	df	MS	*η* ^*2*^
BMI	Age	22.76***	1	1130.73	.017
	Gender	0.73	1	36.23	.001
	Education	0.04	1	1.86	.000
	Eating Profile	10.90***	3	541.30	.024
CIA	Age	65.78***	1	5331.41	.047
	Gender	2.65	1	214.77	.002
	Education	0.14	1	11.27	.000
	BMI	100.05***	1	8108.98	.070
	Eating Profile	9.82***	3	795.57	.022
PHQ-9	Age	39.10***	1	1283.45	.029
	Gender	1.24	1	40.82	.001
	Education	4.28*	1	140.34	.003
	BMI	73.96***	1	2427.93	.053
	Eating Profile	14.35***	3	471.04	.031
Social Eating Anxiety	Age	35.36***	1	286.84	.026
	Gender	0.05	1	0.43	.000
	Education	1.14	1	9.25	.001
	BMI	31.31***	1	253.97	.023
	Eating Profile	19.46***	3	157.81	.042
EDDS	Age	73.96***	1	22.37	.053
	Gender	19.21***	1	5.81	.014
	Education	1.79	1	0.54	.001
	BMI	184.17***	1	55.71	.122
	Eating Profile	9.60***	3	2.90	.021
APEQ	Age	20.72***	1	8.34	.015
	Gender	21.62***	1	8.75	.016
	Education	0.14	1	0.06	.000
	BMI	6.90**	1	2.79	.005
	Eating Profile	48.30***	3	19.55	.099
IES-Uncond	Age	11.10**	1	6.34	.008
	Gender	12.14**	1	6.94	.009
	Education	16.14***	1	9.22	.012
	BMI	19.13***	1	10.93	.014
	Eating Profile	0.49	3	0.28	.001
IES-Phys	Age	17.66***	1	13.60	.013
	Gender	15.55*	1	11.97	.012
	Education	3.14	1	2.42	.002
	BMI	165.32***	1	127.28	.111
	Eating Profile	18.83***	3	14.49	.041
IES-Hung	Age	1.54	1	0.57	.001
	Gender	0.09	1	0.04	.000
	Education	0.60	1	0.22	.000
	BMI	52.32***	1	19.51	.038
	Eating Profile	13.35***	3	4.98	.029

*Note*: *BMI* Body mass index, *CIA* Clinical impairment assessment, *PHQ-9* Patient health questionnaire - 9-item, *EDDS* Eating disorder diagnostic scale, *APEQ* Adult picky eating questionnaire, *IES-Uncond* Intuitive eating scale-Unconditional permission to eat scale, *IES-Phys* Eating for physical vs. emotional reasons scale, *IES-Hung* Eating in response to hunger/satiety scale

* *p* < .05, ** *p* < .01, *** *p* < .001

Results from the ANCOVAs provided convergent and discriminant validity for the *picky eater* profile. As expected, individuals grouped in the *picky eater* profile scored the highest on the APEQ and social eating anxiety in comparison to all other groups. Scores on the EDDS composite score discriminated the *picky eater* profile from the *approaching eater* profile, which appears to capture problematic eating related to chronic overeating and binge eating. The *picky eater* profile was comparable to the *moderate* and *joyful* profiles on the EDDS.

Participants grouped in the *picky eater* profile reported higher scores on the PHQ-9 relative to the *moderate* and *joyful eater* profiles, though their scores were comparable to the *approaching eater* profile. Their scores on the CIA were also higher than individuals in the *moderate eater* profile. In other words, while individuals in the *picky eater* profile reported greater psychosocial impairment, this impairment was most associated with extreme scores in both directions on food approach and avoidance.

As hypothesized, individuals in the *picky eater* profile reported higher scores on the IES-Physical subscale in comparison to the *approaching* and *joyful eater* profiles, suggesting that picky eaters are more likely to eat for physical as opposed to emotional reasons. Individuals grouped in the “picky eater” group also scored lower than the *joyful eater* profile on the IES-Hunger subscale. These results suggest that the *joyful eater* group reports the greatest ability to guide their eating by relying on hunger and satiety cues.

Finally, a Pearson Chi-Square test indicated there were significant differences in the proportional distribution of latent eating profile membership within BMI classification groups, *χ*^*2*^ = 39.81, *p* < .001. As shown in Table 3, the *picky eater* group included a significantly larger proportion of normal weight individuals (52.3%) in comparison to the other eating profiles. The *approaching eater* group included a significantly larger proportion of individuals falling within the obese classification compared to all other groups, and the *picky eater* group included significantly fewer individuals in the obese group compared to the *joyful eater* group but not the *moderate eater* profile.

## Discussion

Recent research suggests that PE behaviors are associated with elevated psychosocial impairment, limited dietary variety and fruit and vegetable intake, and the potential to manifest into food restriction severe enough to lead to the weight, nutritional, and/or psychosocial criteria for a diagnosis of ARFID [42, 43]. Given the relatively high prevalence and potential clinical significance of PE, there is a surprising absence of studies aimed at operationalizing, measuring, and identifying correlates of PE in adults. The present study is one of the first to investigate picky eating in the context of other appetitive traits in adults, with the goal of better understanding the construct of PE.

As hypothesized, LPA identified a *picky eater* profile characterized by low scores on measures food approach and higher scores on food avoidance. The adult *picky eater* profile closely resembled the child profile described by Tharner and colleagues (2014), with one exception: adult picky eaters reported average scores on a measure of slowness in eating, while child picky eaters exhibited high scores on slowness in eating. The divergent results found on this variable may be due to picky eaters increasing their eating speed, or gaining more control over their eating choices, as they age. Indeed, longitudinal research has found that eating slowly significantly decreased in children between the ages of 4 and 10, perhaps because children learn to become more proficient at eating [27]. The sample from the previous child latent profile analysis only included 4-year olds [22]; thus, slowness in eating may not be an important aspect of PE behavior as people age.

The other three profiles that emerged included *moderate eaters* who were characterized by a pattern of moderate food approach and avoidance, *joyful eaters* who were characterized by a moderate pattern of food approach and avoidance but also high food enjoyment, and *approaching eaters* who were characterized by a pattern of high food approach (i.e. high food/eating enjoyment and high responsiveness to eat based on food cues such as smell, hunger, or cognitions about foods) and low food avoidance. These three profiles paralleled the patterns previously identified in children [22]. However, Tharner and colleagues (2014) also identified *avoidant* and *responsive* eater profiles in children, which did not emerge in the current adult sample. Perhaps some eating styles converge as people age, and there may be less variability in approach and avoidant eating patterns in adulthood. Other possible explanations include the differences in methodological approaches. Tharner and colleagues (2014) asked mothers to rate child eating behavior, and mothers may perceive their children to be slow eaters while participants themselves do not. Adults also have significant freedom in food choice that most children do not, which could lead to differences in eating behavior profiles.

In contrast to prior research exploring adult PE, which estimated that approximately 30–35% of adults from community samples report being at least somewhat picky [3, 4], results from the present study found that just 18.1% of adults were classified into the picky eater profile. The lower prevalence in this study may reflect the importance of multifactor assessment when describing PE, as prior research has relied on a single PE item or on responses to scales that assess only PE. Future research should continue to use more precise and multifactor instruments when measuring PE, as broadly-worded single items may overestimate the number of individuals who experience clinically-relevant levels of PE. On the other hand, our PE prevalence of 18.1% is larger than the 5.6% of children previously identified as *fussy eaters*. Given that Tharner and colleagues (2014) also categorized 33.2% of children into a less severe *avoidant eater* group, it may be the case that our latent profile analysis converged on a profile that combined individuals who might have been categorized as either *fussy* or *avoidant* in Tharner and colleagues’ analysis. Additional research is warranted to better understand the relative prevalence of PE in children and adults, and how different measures may overestimate or underestimate the true prevalence of PE.

To support the secondary aim of the study, a series of ANCOVAs provided convergent and divergent validity for an adult *picky eater* profile. As hypothesized, compared to all other profiles, individuals with the *picky eater* profile scored higher on measures of adult PE and social eating anxiety. In comparison to the *moderate* eating profile, those in the *picky eater* profile also scored higher on measures of eating-related impairment and depression, and were more likely to eat based on physiological cues, as opposed to emotional cues. Intuitive eating, based on physiological cues as opposed to emotional factors is considered healthy [36]. Participants in the *picky eating* profile also scored at similar levels as adult picky eaters identified by Wildes and colleagues (2012) on the modified clinical impairment assessment (*M* = 8.9). Furthermore, individuals in the *picky eater* profile reported significantly fewer traditional disordered eating behaviors (i.e., binging, purging, and restrictive behaviors related to shape and weight concerns) in comparison to the *approaching* profile, and similar levels as the *moderate* and *joyful* eaters. While adult PE behaviors can be comorbid with symptoms of other eating disorders, it has also been shown to be a distinct eating pattern [5]. It should also be noted that participants with the *approaching* profile, which appears to reflect overeating and/or disordered eating patterns, reported the lowest scores on a measure of PE. In sum, these findings support the notion that adult PE is similar to PE patterns observed in childhood, and provides support that adult PE indeed represents a unique and measurable pattern of eating behavior.

Perhaps the most illuminating findings were the relationships between BMI classifications and the eating behavior profiles. Results indicated that in comparison to all other eating profiles, picky eaters were significantly more likely to be of a healthy weight and significantly less likely to fall into the obese category relative to the *approaching* and *joyful* profiles. Previous findings using a latent class analysis showed that adult picky eaters were more likely to be of healthy weight in comparison to a disordered eating class and comorbid PE/disordered eating class [5]. However, the present study is the first to establish this association in a nonclinical adult sample, and the first to observe findings similar to those in children; picky eating in combination with unenthusiastic eating and satiety responsiveness is inversely associated with bodyweight [22]. Other investigations of adult PE have not shown differences between PE and non-PE groups, or a relationship between continuous measures of PE and BMI [3, 6, 10, 14, 20]. It appears that PE behavior, when combined with appetitive traits associated with reduced energy intake, may be protective against obesity. On the other hand, given the decreased food variety and decreased fruit and vegetable intake observed in adult picky eaters [8], as well as the psychosocial impairment associated with this eating behavior [5, 6], the implications of this finding for our understanding of the overall health impact of picky eating are unclear.

There are several important limitations to the current study that warrant comment. First, there are several issues related to using MTurk to recruit an online sample. While MTurk samples tend to be more diverse compared to college student samples, a recent systematic review of MTurk samples shows that compared to the general US population a larger proportion of MTurkers are unemployed or underemployed and self-report higher negative emotions and attitudes [41]. In addition, there tend to be high dropout rates [40] as workers may search for HITs that are of topical interest or are financially appealing; leading to self-selection bias. It is possible that individuals who have interest in eating behaviors or eating concerns were less likely to drop out. It is important that these findings are replicated in other samples that may be more generalizable to the US population. The study also relied exclusively on self-report measures, as opposed to structured interviews, to assess eating behavior, disordered eating, and psychosocial constructs. In general, study participants tend to underestimate self-reported BMI and BMI classification, with the obese category being most likely to be misclassified [44]. The reliance of self-reported BMI clearly limits findings in the current study, and future researchers should use measured BMI to more accurately assess the relationship between adult PE and weight.

The present study did not include a measure of dietary intake, which raises issues related to the misclassification of PE. Some individuals included in the *picky eater* profile could be regularly consuming only 3–4 types of food, while others may be consuming 30 to 40. Future research should utilize measures of dietary intake to quantify dietary variety and how it relates to the associations presented in the present study. The study is also cross-sectional, and longitudinal research methods have yet to be used to examine the relationships between adult PE and psychosocial distress and impairment. The cross-sectional nature of the study also limits our understanding of whether or not PE emerged later in life or may be related to other factors that influence BMI. LPA also relies on a certain degree of subjective decision-making, with the objective support of statistical comparisons; thus, arguments could be made to converge on fewer or more profiles. Strengths include the use of a large age-diverse adult sample, and the use of the APEQ, a validated comprehensive measure of adult PE.

## Conclusions

Recent preliminary research into adult PE has highlighted the presence of elevated indicators of psychosocial impairment, and call for further work to operationalize the construct in order to identify potential risk and maintaining factors. The validation of improved instruments to measure PE and identification of associated approach and avoidance eating behaviors was greatly needed, and now researchers can more confidently proceed with longitudinal research investigating the progression of PE-related problems across the lifespan. The present investigation observed a distinct adult PE profile that matches similar eating patterns in children. It appears that difficulties with avoidant eating behavior can, in many cases, carry over into adulthood, and are associated with distress across various domains. However, it is likely that a large portion of individuals who fall into a PE profile do not exhibit clinically significant impairment and distress. There may be distinct groups of picky eaters, perhaps characterized by differing levels of appetite and enthusiasm for eating, with different obesity risk based on different energy intake. Future studies should use latent profile analysis to identify meaningful subcategories within samples of self-identified adult picky eaters.

## References

[1] Dovey TM, Staples PA, Gibson EL, Halford JCG (2008). Food neophobia and ‘picky/fussy’ eating in children: a review. Appetite.

[2] Taylor CM, Wernimont SM, Northstone K, Emmett PM (2015). Picky/fussy eating in children: review of definitions, assessment, prevalence and dietary intakes. Appetite.

[3] Kauer J, Pelchat ML, Rozin P, Zickgraf HF (2015). Adult picky eating. Phenomenology, taste sensitivity, and psychological correlates. Appetite.

[4] Zickgraf HF, Franklin ME, Rozin P. Adult picky eaters with symptoms of avoidant/restrictive food intake disorder: comparable distress and comorbidity but different eating behaviors compared to those with disordered eating symptoms. J Eat Disord. 2016;4:1-11. 10.1186/s40337-016-0110-6 PMC5086050 27800160

[5] Wildes JE, Zucker NL, Marcus MD (2012). Picky eating in adults: results of a web-based survey. Int J Eat Disord.

[6] Ellis JM, Galloway AT, Webb RM, Martz DM (2017). Measuring adult picky eating: the development of a multidimensional self-report instrument. Psychol Assess.

[7] American Psychiatric Association. Diagnostic and statistical manual of mental disorders (DSM-5®). Arlington: American Psychiatric Pub; 2013.

[8] Zickgraf HF, Schepps K (2016). Fruit and vegetable intake and dietary variety in adult picky eaters. Food Qual Preference.

[9] Wardle J, Guthrie CA, Sanderson S, Rapoport L (2001). Development of the children’s eating behaviour questionnaire. J Child Psychol Psychiatry.

[10] Hunot C, Fildes A, Croker H, Llewellyn CH, Wardle J, Beeken RJ (2016). Appetitive traits and relationships with BMI in adults: development of the adult eating behaviour questionnaire. Appetite.

[11] Galloway AT, Lee Y, Birch LL (2003). Predictors and consequences of food neophobia and pickiness in young girls. J Am Diet Assoc.

[12] Haszard JJ, Skidmore PML, Williams SM, Taylor RW (2015). Associations between parental feeding practices, problem food behaviours and dietary intake in New Zealand overweight children aged 4-8 years. Public Health Nutr.

[13] Brown CL, Vander Schaaf EB, Cohen GM, Irby MB, Skelton JA (2016). Association of picky eating and food neophobia with weight: a systematic review. Child Obes.

[14] Mallan KM, Fildes A, de la Piedad GX, Drzezdzon J, Sampson M, Llewellyn C (2017). Appetitive traits associated with higher and lower body mass index: evaluating the validity of the adult eating behaviour questionnaire in an Australian sample. Int J Behav Nutr Phys Act.

[15] Antoniou EE, Roefs A, Kremers SPJ (2016). Picky eating and child weight status development: a longitudinal study. J Hum Nutr Diet.

[16] Taylor CM, Steer CD, Hays NP, Emmett PM. Growth and body composition in children who are picky eaters: a longitudinal view. Eur J Clin Nutr. July 2018. 10.1038/s41430-018-0250-7. 10.1038/s41430-018-0250-7 PMC6215483 29995831

[17] Zucker N, Copeland W, Franz L, et al. Psychological and psychosocial impairment in preschoolers with selective eating. Pediatrics. 2015; peds.2014–2386. 10.1542/peds.2014-2386 PMC4552088 26240213

[18] Mascola AJ, Bryson SW, Agras WS (2010). Picky eating during childhood: a longitudinal study to age 11 years. Eat Behav.

[19] Ellis JM, Schenk RR, Galloway AT, Zickgraf HF, Webb RM, Martz DM (2018). A multidimensional approach to understanding the potential risk factors and covariates of adult picky eating. Appetite.

[20] Zickgraf HF, Ellis JM (2017). Initial validation of the nine item avoidant/restrictive food intake disorder screen (NIAS): a measure of three restrictive eating patterns. Appetite.

[21] de Barse LM, Tiemeier H, Leermakers ETM, Voortman T, Jaddoe VWV, Edelson LR (2015). Longitudinal association between preschool fussy eating and body composition at 6 years of age: the generation R study. Int J Behav Nutr Phys Act.

[22] Tharner A, Jansen PW, Kiefte-de Jong JC, Moll HA, van der Ende J, Jaddoe VWV (2014). Toward an operative diagnosis of fussy/picky eating: a latent profile approach in a population-based cohort. Int J Behav Nutr Phys Act.

[23] Sandvik P, Ek A, Somaraki M, Hammar U, Eli K, Nowicka P (2018). Picky eating in Swedish preschoolers of different weight status: application of two new screening cut-offs. Int J Behav Nutr Phys Act.

[24] Ellis JM, Galloway AT, Zickgraf HF, Whited MC (2018). Picky eating and fruit and vegetable consumption in college students. Eat Behav.

[25] Van Tine ML, McNicholas F, Safer DL, Agras WS (2017). Follow-up of selective eaters from childhood to adulthood. Eat Behav.

[26] Shepard DN, Chandler-Laney PC (2015). Prospective associations of eating behaviors with weight gain in infants. Obesity.

[27] Ashcroft J, Semmler C, Carnell S, van Jaarsveld CHM, Wardle J (2008). Continuity and stability of eating behaviour traits in children. Eur J Clin Nutr.

[28] Carnell S, Haworth CMA, Plomin R, Wardle J (2008). Genetic influence on appetite in children. Int J Obes.

[29] Dubois L, Diasparra M, Bédard B (2013). Genetic and environmental influences on eating behaviors in 2.5- and 9-year-old children: a longitudinal twin study. Int J Behav Nutr Phys Act.

[30] Llewellyn CH, van Jaarsveld CHM, Johnson L, Carnell S, Wardle J (2010). Nature and nurture in infant appetite: analysis of the Gemini twin birth cohort. Am J Clin Nutr.

[31] Paolacci G, Chandler J (2014). Inside the Turk: understanding Mechanical Turk as a participant pool. Curr Dir Psychol Sci.

[32] Kroenke K, Spitzer RL, Williams JB (2001). The PHQ-9: validity of a brief depression severity measure. J Gen Intern Med.

[33] Bohn K, Doll HA, Cooper Z, O’Connor M, Palmer RL, Fairburn CG (2008). The measurement of impairment due to eating disorder psychopathology. Behav Res Ther.

[34] Stice E, Fisher M, Martinez E (2004). Eating disorder diagnostic scale: additional evidence of reliability and validity. Psychol Assess.

[35] Stice E, Ragan J (2002). A preliminary controlled evaluation of an eating disturbance psychoeducational intervention for college students. Int J Eat Disord..

[36] Tylka T (2006). Development and psychometric evaluation of a measure of intuitive eating. J Counseling Psych.

[37] Core Team R (2015). R: a language and environment for statistical computing.

[38] Scrucca L, Fop M, Murphy TB, Raftery AE (2016). Mclust 5: clustering, classification and density estimation using Gaussian finite mixture models. R J.

[39] Swanson SA, Lindenberg K, Bauer S, Crosby RD (2012). A Monte Carlo investigation of factors influencing latent class analysis: an application to eating disorder research. Int J Eat Disord.

[40] Zhou H, Fishbach A (2016). The pitfall of experimenting on the web: how unattended selective attrition leads to surprising (yet false) research conclusions. J Pers Soc Psychol.

[41] Keith MG, Tay L, Harms PD. Systems perspective of Amazon Mechanical Turk for organizational research: review and recommendations. Front Psychol. 2017;8. 10.3389/fpsyg.2017.01359. 10.3389/fpsyg.2017.01359 PMC5550837 28848474

[42] Nicely TA, Lane-Loney S, Masciulli E, Hollenbeak CS, Ornstein RM (2014). Prevalence and characteristics of avoidant/restrictive food intake disorder in a cohort of young patients in day treatment for eating disorders. J Eat Disord.

[43] Norris Mark L., Spettigue Wendy, Hammond Nicole G., Katzman Debra K., Zucker Nancy, Yelle Katie, Santos Alexandre, Gray Madeline, Obeid Nicole (2017). Building evidence for the use of descriptive subtypes in youth with avoidant restrictive food intake disorder. International Journal of Eating Disorders.

[44] Gosse MA (2014). How accurate is self-reported BMI?. Nutr Bull.

